# Trace Lithium for Suicide Prevention and Dementia Prevention: A Qualitative Review

**DOI:** 10.3390/ph17111486

**Published:** 2024-11-05

**Authors:** Takeshi Terao, Hirofumi Hirakawa, Masaaki Muronaga, Toshihiko Izumi, Kentaro Kohno

**Affiliations:** Department of Neuropsychiatry, Oita University Faculty of Medicine, Daigaoka 1-1, Hasama-machi, Yufu 879-5593, Oita, Japan; hira-hiro@oita-u.ac.jp (H.H.); m-muronaga@oita-u.ac.jp (M.M.); tshhkizm@oita-u.ac.jp (T.I.); kentarokohno@oita-u.ac.jp (K.K.)

**Keywords:** lithium, suicide prevention, dementia, trace elements

## Abstract

**Background**: Anti-manic effects of lithium and the nature of trace element in lithium were first observed in 1949. In this review, we explore the potential effects of trace lithium in the prevention of suicide and dementia. **Methods**: This is a qualitative and non-systematic review. **Results**: While most studies to date have been cross-sectional, limiting the establishment of causal relationships, the potential benefits of trace lithium for suicide prevention and dementia prevention are notable, especially in the absence of radical treatments for suicide and dementia. Furthermore, trace lithium appears to lack many of the adverse effects associated with so-called therapeutic lithium levels. **Conclusions**: The present findings suggest that trace lithium may be associated with lower suicide rates and reduced dementia rates. Probably, trace lithium may inhibit testosterone and thereby mitigate aggression and impulsivity and decrease suicide. Also, trace lithium may inhibit GSK-3β and thereby lower amyloid β and tau hyperphosphorylation and inhibit pro-inflammatory cytokines such as IL 6 and IL 8 and thereby mitigate inflammation, whereas trace lithium may promote BDNF and neurogenesis in the general population.

## 1. Introduction

For the first time, Cade [1] identified the therapeutic effects of lithium effects for bipolar disorder, noting, “There is no doubt that in mania patients improvement had closely paralleled (lithium) treatment” and “The quietening effect on restless non-manic psychotics is additional strong evidence of the efficacy of lithium salts, especially as such restlessness returned on cessation of (lithium) treatment”. Subsequent studies have repeatedly confirmed lithium’s mood-stabilizing properties in bipolar disorder [2,3,4], as well as its efficacy in augmentation therapy for treatment-resistant major depression [5,6,7]. Notably, several investigations have demonstrated lithium’s anti-suicidal effects [8,9] and potential anti-dementia properties [10,11,12]. Cade [1] also speculated, “Lithium may well be an essential trace element. It is widely distributed, has been detected in sea-water and in many spring and river waters, in the ash of many plants, and in animal ash”.

As for the clinical use of lithium for bipolar disorder, recently Shuy et al. [13] reported that lithium salts were prescribed in 27.3% of 2139 patients suffering from bipolar disorder (52.3% women, mean age 42.4 years), varying among regions from 3.20% to 59.5%. Associated with lithium treatment were male sex, the presence of euthymia or mild depression, and a history of seasonal mood change. Other mood stabilizers were usually given with lithium, often at relatively high doses. Lithium use was associated with a newly emerging and dose-dependent risk of tremors as well as a risk of hypothyroidism. Shuy et al. [13] found no significant differences in rates of clinical remission or of suicidal behavior whether treatment included lithium or not. As for the apparent lack of an association of lithium treatment with expected reduction of suicidal behavior, Shuy et al. [13] consider the possibility of the short exposure of lithium and the relative infrequency of suicide acts.

In this review, we focus on the effects of trace lithium, defined as very low levels of lithium far below the therapeutic levels commonly used in routine psychiatric practice. This review is not intended to be a systematic or quantitative analysis but rather a qualitative exploration of the potential effects of trace lithium in the prevention of suicide and dementia prevention. The aim of this review is to reconfirm these effects and to specifically suggest the mechanisms at low lithium levels.

## 2. Lithium for Suicide Prevention

Wang et al. [14] showed that among 1647 patients with bipolar disorder across 13 Asian countries, the doses of mood stabilizers averaged 584 (confidence interval, 565–603 lithium-equivalent mg/day), and 13.1% of the patients given greater than 900 mg/day were younger, male, currently hospitalized, not currently depressed, and reported lifetime suicidal ideation in comparison to those given lower doses. Probably, higher doses of lithium may be required to treat lifetime suicidal ideation in some patients.

Conversely, since the initial report of a significant inverse association between lithium levels in drinking water and suicide rates across 27 Texas counties [15], numerous studies have investigated the association between lithium levels in drinking water and suicide rates, including our own studies [16,17,18]. For instance, Ohgami et al. [16] measured lithium levels in drinking water in the Oita prefecture of Japan and calculated the suicide standardized mortality ratio (SMR). Their findings indicated that lithium levels were significantly and inversely associated with suicide SMRs, suggesting that even trace amounts of lithium levels in drinking water may help reduce suicide risk in the general population. In contrast, Kabacs et al. [19] found no significant association between lithium in drinking water and suicide rates in the East of England. Meanwhile, Kapusta et al. [20] reconfirmed a significantly inverse association between lithium levels in drinking water and suicide SMRs in Austria, even after adjusting for socioeconomic factors.

Since these studies, further epidemiological investigations have continued to explore this association. Recently, Kugimiya et al. [21] conducted an extensive study across all 808 cities and wards in Japan (785 cities from 46 prefectures and 23 wards of Tokyo), investigating the association between lithium levels in drinking water and suicide SMRs during the 7 years from 2010 to 2016. Using multiple regression analyses adjusted for the size of each population size and other relevant factors, they found significantly inverse associations of lithium levels with total and male SMRs but not with female SMRs [21]. These findings further strengthen the evidence supporting an inverse association between lithium levels in drinking water and suicide rates, particularly in men. Finally, recent meta-analyses [22,23,24] have confirmed that higher lithium levels in drinking water may be associated with lower suicide rates.

With regard to the anti-suicidal effects of therapeutic levels of lithium in clinical settings, a recent meta-analysis [25] reported that the pooled suicide rate was 0.2% for individuals randomized to lithium and 0.4% for those receiving a placebo or treatment as usual, which was not a statistically significant difference [odds ratio = 0.42, 95% confidence interval (CI) 0.01–4.5]. However, a recent reanalysis [9] appropriately criticized the methodological flaws in that meta-analysis [25] and revealed a larger effect size. The reanalysis showed that the pooled suicide rate was 0.3% for individuals randomized to lithium and 1.69% for those receiving placebo or treatment as usual, with an odds ratio of 0.25 (95% CI 0.08–0.83), suggesting a 75% reduction in suicide completion associated with lithium drugs in patients with mood disorders.

**Figure 1:**
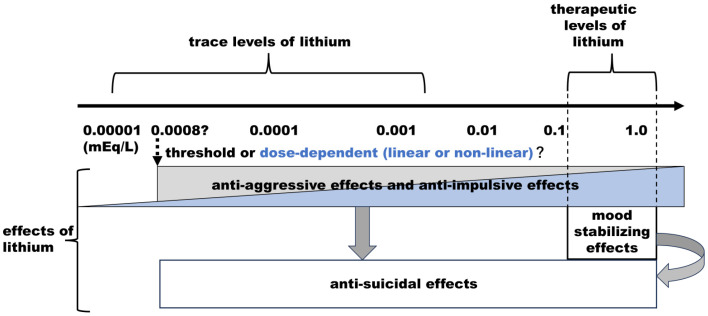
The effects of lithium from trace levels to therapeutic levels. Trace lithium levels primarily produce anti-aggressive/anti-impulsive effects, contributing to anti-suicidal outcomes, while therapeutic levels of lithium add mood-stabilizing effects. The threshold for anti-aggressive/anti-impulsive effects may occur at 0.0008 mEq/L as a threshold, or the effect could follow a dose–response pattern (linear or non-linear).

It is challenging to directly compare the lithium effects of trace lithium levels with therapeutic levels due to differences in the populations studied, namely, the general population versus patients with mood disorders. However, the contrast between a 58% reduction in suicide completion in the general population associated with trace levels of lithium levels [22] and a 75% reduction of suicide completion in patients with mood disorders associated with therapeutic levels of lithium levels [9] suggests that higher levels of lithium may exert stronger anti-suicidal effects. As illustrated in Figure 1, trace lithium levels may predominantly elicit anti-aggressive and anti-impulsive effects, which in turn could contribute to reducing suicide risk. On the other hand, therapeutic levels of lithium levels appear to generate both mood-stabilizing effects and anti-aggressive and anti-impulsive effects, enhancing their overall anti-suicidal impact. This could explain why therapeutic levels of lithium may offer more potent anti-suicidal effects than trace levels of lithium.

Epidemiological studies investigating the association between lithium levels in drinking water and suicide rates may be subject to ecological fallacy. To address this, we further examined the association between trace lithium levels and suicide attempts at the individual level [26,27,28]. These participants were not receiving lithium therapy and were presumed to ingest trace lithium through drinking water and food sources. The most recent study by Izumi et al. [28] explored the effects of naturally absorbed trace lithium alongside unsaturated fatty acids such as eicosapentaenoic acid (EPA), docosahexaenoic acid (DHA), and arachidonic acid (AA) on deliberate self-harm and suicide attempts in 234 patients. The findings indicated that higher serum lithium levels were significantly associated with fewer suicide attempts and fewer deliberate self-harm, even after adjusting for age, sex, and unsaturated fatty acids (EPA, DHA, or AA). Mean serum lithium levels in the suicide attempt group, self-harm group, and control group were 4.04, 4.37, and 5.62 μg/L, respectively, corresponding to 0.00058, 0.00063, and 0.00081 mEq/L [28]. If the association between lithium levels and anti-aggressive/anti-impulsive effects has a cut-off value (a threshold, it may lie around 0.0008 mEq/L (gray section of Figure 1). Alternatively, there may be a dose–response relationship between serum lithium levels (blue section of Figure 1). Currently, it is unclear whether this association follows a threshold or a dose–response pattern.

Although our studies focused on suicide attempters, Ando et al. [29] examined suicide completers by measuring lithium levels in the aqueous humor, which remains relatively stable after death. Their findings revealed that lithium levels in the aqueous humor of 12 suicide completers (mean 0.50 μg/L) were significantly lower than those in 16 controls with non-suicidal deaths (mean 0.92 μg/L). These results suggest that similar to suicide attempters, suicide completers may have significantly lower lithium levels in the body compared to non-suicidal controls. This represents important evidence for the inverse association between lithium levels and suicide completion, as strictly speaking, suicide attempts and suicide completion are different.

One of the animal studies [30] showed that lithium decreased shock-induced aggression in mice. One potential mechanism by which low-dose lithium may prevent suicide prevention is its effect on testosterone levels, which are associated with aggression and impulsivity. Testosterone is the primary androgen hormone responsible for male sexual development and the maintenance of male secondary sexual characteristics. In men, testosterone is predominantly secreted by Leydig cells in the testes, though a smaller amount of testosterone is also produced by the adrenal glands. In women, lesser quantities of testosterone are secreted by the adrenal glands and ovaries [31]. It has been suggested that testosterone modulates social-emotional behavior in men through prefrontal-amygdala functional connectivity [31]. Sher et al. [32,33] also reported that higher testosterone levels were linked with increased suicidality in both men and women, with testosterone levels generally being much higher in men. Furthermore, lithium administration has been shown to reduce testosterone levels, suggesting that lithium may lower suicidality, particularly in men, by decreasing testosterone levels [34]. This points to the possibility that lithium’s inhibition of testosterone may reduce aggression and impulsivity, thus contributing to suicide prevention.

**Figure 2:**
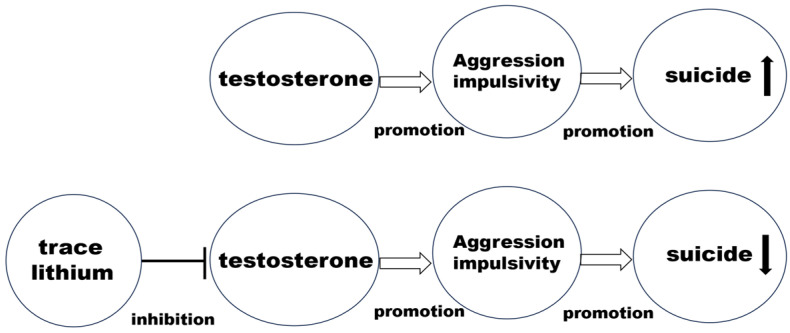
Lithium against testosterone, aggression/impulsivity, and suicide. Trace lithium may inhibit testosterone, which may decrease aggression and impulsivity, leading to suicide prevention.

With respect to trace lithium, an observational study conducted in China followed 796 college students who were recruited from June 2013 (baseline) to 2014 to investigate the effects of exposure to metal/metalloid elements on male fertility [35]. During both phases, semen and blood samples were collected to assess semen quality and levels of six sex hormones. One key finding was that the median urinary lithium levels (μg/L) were 16.5 at baseline and 16.69 at follow-up, while median serum testosterone levels (nmol/L) were 4.3 at baseline and 4.0 at follow-up. After adjusting for confounding factors, urinary lithium levels were found to be significantly and inversely associated with serum testosterone [35], suggesting that trace lithium may be inversely associated with serum testosterone levels [35]. These results suggest that even trace lithium may also reduce testosterone levels, which in turn could lower aggression and impulsivity, leading to suicide prevention, as illustrated in Figure 2.

## 3. Lithium for Dementia Prevention

Nunes et al. [36] randomized 113 patients with Alzheimer’s disease (AD) and Mini Mental State Evaluation (MMSE) scores ranging from 9 to 21 to receive either lithium (300 μg/day; n = 58) or placebo (n = 55) treatments in a 15-month, randomized, placebo-controlled, double-blind trial. The lithium group demonstrated no decline in their performance on the MMSE scores, whereas the placebo group showed a decline, suggesting a potential neuroprotective effect of trace lithium on AD progression [36].

Regarding epidemiological studies, Kessing et al. [37] examined the potential association between lithium levels in drinking water and the incidence of dementia in the general population of Denmark. They found that higher lithium levels in drinking water were nonlinearly associated with a reduced incidence of dementia. In contrast, Parker et al. [38] reported no significant association between higher lithium levels in drinking water and the prevalence of dementia. These conflicting results highlight the variability in epidemiological studies. Additionally, Fajardo et al. [39] investigated AD mortality and demonstrated a significantly inverse association between lithium levels in drinking water and changes in AD mortality, suggesting that trace lithium may influence AD mortality rates.

Recently, Muronaga et al. [40] investigated the association between lithium levels in drinking water and the prevalence of AD across all 808 cities and wards in Japan. Their study demonstrated a significantly inverse association between lithium levels and the prevalence of AD in females but not in males or the overall population after adjusting for several factors associated with dementia. These findings suggest that higher lithium levels in drinking water may be associated with a low prevalence of AD in females, though no such association was observed in males, which contrasts with our previous findings related to suicide rates [21].

**Figure 3:**
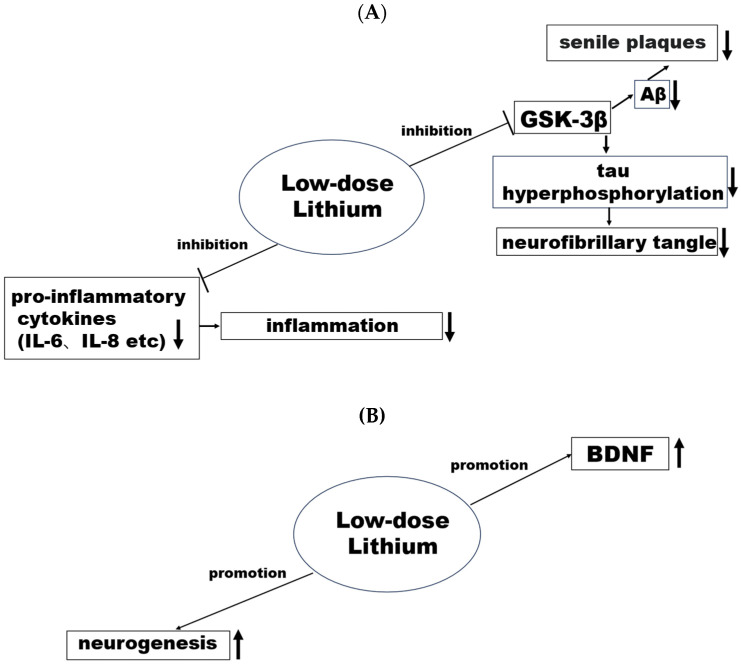
The mechanisms of trace lithium for dementia prevention. (**A**): lithium inhibition, (**B**): lithium promotion. Low-lithium may inhibit Glycogen Synthase Kinase-3β (GSK-3β) and pro-inflammatory cytokine (IL-6, IL-8, etc.) and promote Brain-derived Neurotrophic Factor (BDNF) and neurogenesis.

With regard to the mechanisms of low-dose lithium for dementia prevention, several factors have been proposed in preclinical studies [41]. For example, as shown in Figure 3A, low-dose lithium may inhibit Glycogen Synthase Kinase-3β (GSK-3β) [42,43] and thereby decrease amyloidβ_42_ levels [42] and senile plaques [43,44]. Also, low-dose lithium may inhibit pro-inflammatory cytokine (IL-6, IL-8, etc.) [45,46] and neuroinflammation. Moreover, as shown in Figure 3B, low-dose lithium may promote Brain-derived Neurotrophic Factor (BDNF) [44,47] and neurogenesis [42,44]. In contrast to the mechanism of lithium for suicide prevention, there are many acting sites of lithium for dementia prevention, although there may be many unrevealed acting sites of lithium for suicide prevention.

## 4. New Lithium Product

Nanolithium is an experimental product utilizing a novel drug-delivery technology (Aonys^®^, MEDESIS PHARMA, Baillargues, France) that optimizes its bioavailability while minimizing its toxicity profile [48].

The therapeutic doses of lithium used in Nanolithium are more than 50 times lower than the minimal doses used in traditional lithium salts. GSK-3β inhibition is believed to be central to Nanolithium’s mechanism of action, leading to a reduction in the production of toxic amyloid plaques and a decrease in tau hyperphosphorylation, which may potentially improve both neuropsychiatric symptoms and cognitive decline. Currently, several clinical studies are underway [48].

## 5. Discussion

Post and Rybakowski [49] depicted the multiple assets of lithium beyond its anti-manic effects, which were demonstrated in recent years as follows.

(1) Diagnosis: Lithium prevents unipolar and bipolar depression.
(2) Suicide: Lithium prevents suicides in patients and in the general population. Lithium prevents suicide in the general population at minuscule doses in the water supply.
(3) Augmentation: Lithium enhances the effects of other mood stabilizers and atypical antipsychotics.
(4) Hippocampus: Lithium increases the volume of the hippocampus.
(5) Gray/white matter: Lithium prevents or reverses cortical gray matter and white matter tract deficits.
(6) Circadian rhythm: Lithium normalizes circadian rhythm abnormalities and has immune-modulating effects.
(7) Immune system: Lithium has immune-modulating effects.
(8) Telomeres: Lithium increases the length of telomeres shortened by episodes, stress, and aging.
(9) Memory: Lithium prevents or slows memory deterioration in mild cognitive impairment over one year.
(10) Dementia: Lithium reduces the incidence of a diagnosis of dementia in old age.
(11) Life expectancy: Lithium prolongs life expectancy and reduces the incidence of all-cause mortality.
(12) All-cause mortality: Lithium reduces the incidence of all-cause mortality.

Among these effects, “Lithium prevents suicide in the general population at minuscule doses in the water supply.” and “Lithium prevents or slows memory deterioration in mild cognitive impairment over one year. Lithium reduces the incidence of a diagnosis of dementia in old age.” match the present findings.

The present findings suggest that trace lithium may be associated with lower suicide rates and reduced dementia rates, although most studies have been cross-sectional, and causality cannot be definitively established. Nonetheless, the potential value of trace lithium appears significant, as there is currently no definitive treatment for suicide prevention or dementia, and trace lithium is likely to avoid the side effects associated with so-called therapeutic lithium levels.

Regarding the experimental drug containing trace lithium named Nanolithium [48], it is expected that the ongoing randomized, placebo-controlled, parallel-group studies can clarify its effects on Alzheimer’s disease. Additionally, it is plausible that Nanolithium may reduce suicide attempts and completions, warranting further investigation. Notably, in Japan, one psychiatrist with extensive experience has prescribed 1 to 2 mg of lithium alongside powdered stomach medication for young psychiatric patients [50]. While the possibility of a placebo effect cannot be ruled out, this approach represents a much simpler treatment compared to Nanolithium.

With regard to the role of trace lithium as an essential trace element [51,52], essential trace elements are generally defined as dietary minerals required in very minute quantities for the proper growth, development, and physiological functioning of an organism. [50]. Regrettably, the available data on trace lithium’s role in human growth, development, and physiology are limited. Nonetheless, a deficiency of trace lithium may contribute to difficulties in controlling aggression and impulsivity, potentially leading to self-harm and/or suicide. Additionally, in line with Schrauzer and Shrestha [15], Kohno et al. [53] demonstrated that lithium levels in drinking water were significantly and inversely associated with crime rates, suggesting that trace lithium may influence aggressiveness and impulsivity toward others as well as oneself.

## 6. Limitations

This is a qualitative and non-systematic review. As such, the references were deviated to the authors’ preference, although we tried to collect references as much as fairly. Also, the quality of the references was various due to different sample sizes and methodologies.

## 7. Conclusions

The present findings suggest that higher trace lithium levels may be associated with lower suicide rates and reduced dementia rates. Probably, trace lithium may inhibit testosterone and thereby mitigate aggression and impulsivity and decrease suicide. Also, trace lithium may inhibit GSK-3β and thereby lower amyloid β and tau hyperphosphorylation, and inhibit pro-inflammatory cytokines such as IL 6 and IL 8 and thereby mitigate inflammation, whereas trace lithium may promote BDNF and neurogenesis in the general population.

## 8. Future Directions

If trace lithium levels are confirmed to be effective for suicide prevention and dementia prevention, it is necessary to clarify whether the association between lithium levels and its anti-suicidal effects and anti-dementia effects follows a dose–response pattern—whether it is linear, non-linear, or characterized by a threshold effect. Eventually, prospective and multicenter studies are warranted to confirm that trace lithium can be effective for suicide prevention and for dementia prevention. Also, further research on pharmacogenetic testing [54] is required for suicide prevention and dementia prevention.
